# Prediction of ultimate load capacity of demountable shear stud connectors using machine learning techniques

**DOI:** 10.1038/s41598-025-15711-4

**Published:** 2025-08-21

**Authors:** Ahmed I. Saleh, Nabil S. Mahmoud, Fikry A. Salem, Mohamed Ghannam

**Affiliations:** 1 Civil Engineering Department, Faculty of Engineering, Delta University for Science and Technology, Gamasa, Egypt; 2 Structural Engineering Department, Faculty of Engineering, Mansoura University, Mansoura, Egypt

**Keywords:** Demountable shear stud connectors, Ultimate shear capacity prediction, Linear regression, Ridge, Lasso, K-Nearest neighbors (KNN), Support vector regression (SVR), Decision tree, Random forest, XGBoost, Engineering, Civil engineering

## Abstract

This study investigates the use of machine learning (ML) models to predict the ultimate load capacity of demountable shear connectors in steel–concrete composite structures. A dataset of 239 experimental and numerical records was assembled, incorporating critical features such as bolt diameter, bolt yield and ultimate strengths, concrete and grout compressive strengths, and multiple interfacial friction coefficients. Eight supervised ML algorithms were evaluated: Linear Regression, Ridge, Lasso, K-Nearest Neighbors (KNN), Support Vector Regression (SVR), Decision Tree, Random Forest, and XGBoost. Model performance was assessed using R², Mean Absolute Error (MAE), and Mean Squared Error (MSE). Among these, XGBoost and Random Forest delivered the best predictive accuracy, with R² values of 0.9477 and 0.9255, respectively, outperforming other methods across all evaluation metrics. SHAP analysis was employed to explain model behavior and identify the most influential features. The analysis revealed that bolt diameter, steel ultimate strength, and bolt-to-concrete friction were the most impactful predictors of shear capacity. These insights were supported by feature importance heatmaps generated from the top-performing models. Despite the robust performance of advanced algorithms, the study acknowledges challenges such as data imbalance and minimal influence of certain parameters, which may affect model generalizability.

**Supplementary Information:**

The online version contains supplementary material available at 10.1038/s41598-025-15711-4.

## Introduction

Steel–concrete composite beams are widely used in modern construction due to their exceptional strength, stiffness, and structural efficiency. These systems rely on shear connectors to ensure effective force transfer between the steel and concrete components, enabling them to behave as a single structural unit. Traditionally, welded headed studs have served this purpose. However, their permanent nature complicates disassembly, recycling, and reuse, especially at the end of a structure’s life, often resulting in mixed waste and lost material value^1^. In response, researchers have introduced demountable shear connectors, which employ mechanical fastening methods—such as bolts, threaded connections, or friction-grip systems—to allow for easier disassembly, material recovery, and reduced waste^2–4^. These connectors align with the principles of the circular economy, which emphasize design for disassembly, extended material usability, and recyclability. Furthermore, traditional welded systems rely on energy-intensive installation, contributing to the construction sector’s high carbon emissions, a sector already responsible for approximately 30% of global solid waste and 39% of energy-related CO₂ emissions^5^.

Beyond sustainability, demountable connectors offer practical advantages, especially in modular construction, where prefabricated parts are assembled and disassembled quickly, reducing labor, construction time, and waste^2^. Studies have confirmed that bolted connectors can match or exceed welded studs in terms of ductility, load-slip performance, and strength, making them suitable for demanding applications such as high-rise buildings and bridges^6^. A growing body of research supports these findings. Ataei et al.^7^ tested bolted shear connectors in thin-walled beams, demonstrating improved ductility and distinct failure modes. Fahmy et al.^8^ conducted experimental and numerical studies on high-strength demountable connectors, evaluating the effects of bolt geometry, grout strength, preloading, and material properties. Their results highlighted the reusability, strength, and stiffness of bolted systems and introduced improved predictive equations.

Further studies have proposed new design models. Ataei^9^ introduced design equations accounting for bolt clearance, preload, and material interaction, offering improved prediction accuracy over existing standards. Similarly, Kwon et al.^10^ proposed retrofitting strategies using post-installed bolted connectors, while Pavlović et al.^11^ demonstrated comparable shear resistance between bolted and welded connectors, emphasizing the dismantling potential of bolted systems.

Studies by Lam and Dai^12^ and Lee and Bradford^13^ focused on eco-friendly materials, such as geopolymer concrete combined with bolted systems, achieving high ductility and CO₂ reduction. Rehman et al.^14^confirmed that such systems met Eurocode 4^15^ requirements for ductility and load capacity. Recent works by Suwaed and Karavasilis^16^Lam et al.^17^and Patel et al.^18^ further validated the structural and environmental benefits of blind bolts and demountable connectors in composite and precast systems. In addition, Kozma et al.^19^Loqman et al.^20^and Hosseini et al.^21^ confirmed that demountable connectors not only allow for deconstruction and reuse but also provide higher fatigue resistance and slip capacity than welded studs. Suwaed^22^ introduced a friction-based, high-strength bolted connector that achieved high shear resistance and rapid disassembly.

Recent studies have incorporated advanced numerical modeling to validate experimental results. Wang et al.^23^ used FE analysis to study high-strength bolted connectors under inverse push-off loading, while Csillag and Pavlović^24^ explored blind-bolted and SRR connectors in FRP decks. Hosseinpour et al.^25^ verified the suitability of bolted connectors in thin-walled cold-formed beams, meeting Eurocode 4 ductility standards. A variety of innovative connector types have emerged: the lockbolt (LB-DSC) with grout-filled tubes and a conical lug^26^Y-shaped bolted connectors with tapered nuts (Jung et al. 2022), and double-nut friction-grip connectors^27^ Song et al.^28^ tested tapered iron bolt (TIB) systems and proposed new design formulations. Liu et al.^29^ and Jakovljević et al.^30^ confirmed the adaptability of single-nut embedded connectors and the reliability of demountable connections across precast and cast-in-place systems. Furthermore, Fang^31^ evaluated high-strength bolted connectors in UHPC, confirming their superior behavior. Fahmy et al.^8^ and Ataei et al.^4^ used a combination of experimental and FEM data to propose predictive models for load capacity and stiffness in cold-formed composite beams. Yu and Kim^32^ analyzed how stud shear connectors behave in steel–UHPC composite structures, especially when using short studs. They improved existing models by factoring in stud head rotation and plastic deformation, which helped better match experimental results. Their study showed that both UHPC strength and stud size have a strong impact on shear performance, offering clearer guidance for designing more efficient composite systems.

Although machine learning has demonstrated strong potential in forecasting the shear behavior of connectors in steel–concrete composite systems, several limitations have been identified in previous research. A common issue is the reliance on small or unbalanced datasets, which restricts the models’ performance across a range of structural conditions. The practical usefulness of these models is often reduced by the neglect or oversimplification of critical factors such as cyclic loading, complex failure mechanisms, and environmental effects. Furthermore, many approaches lack transparency, providing predictions without clearly explaining the contribution of input variables to the final outcome, making them less accessible for engineers in real-world design applications. Additionally, few studies integrate both numerical and experimental data to enhance model accuracy.

Several studies have demonstrated the effectiveness of machine learning in predicting the shear behavior of stud connectors in steel–concrete composite structures. Zhu and Farouk^33^ employed artificial neural networks enhanced by particle swarm optimization to predict the shear resistance of grouped stud connectors, outperforming conventional design codes based on 232 push-out tests. Similarly, Lee et al.^34^ evaluated different Machine Learning (ML) models, finding that artificial neural networks provided the most accurate predictions. Their use of global sensitivity analysis identified stud diameter, concrete strength, and stud yield strength as key influencing factors. Zhou et al.^35^ further applied various machine learning models, including eXtreme Gradient Boosting (XGBoost) and Random Forest, to predict the shear strength of headed studs in steel–UHPC systems using 194 test samples. SHapley Additive explanations (SHAP) analysis was used to interpret feature importance, and a user-friendly tool was developed to support engineering applications.

In related work, Li et al.^36^ proposed an ensemble stacking model combining XGBoost, LightGBM, and ExtraTrees to estimate the shear capacity of Perfobond Rib (PBL) connectors using 245 test results. Their model achieved high accuracy (R² = 0.92) and interpretability, with concrete strength, rebar count, and rebar yield strength identified as the most significant features via SHAP analysis. Taffese et al.^37^ also developed an explainable AI model using LightGBM and SHAP to predict slip behavior at the steel–UHPC interface, highlighting stud diameter and spacing as dominant factors. These studies^33–37^ emphasize the growing integration of interpretable machine learning techniques in structural prediction tasks, providing both accurate results and valuable design insights.

For fatigue behavior, Kang et al.^38^ introduced a Gaussian Process Regression model trained on a large fatigue test dataset to predict the life of stud connectors in steel–concrete bridges. Their approach outperformed traditional design codes and provided insights into the most influential fatigue-related variables through explainable AI techniques.

He et al.^39^ studied machine learning applications in the design, optimization, and evaluation of steel–concrete composite structures, providing a more comprehensive viewpoint. In addition to highlighting the effectiveness of ML models in prediction and damage assessment tasks, the review also identified many significant drawbacks, including a lack of data, a lack of model transparency, and a lack of real-world validation.

Despite these advancements, most existing models are empirical and limited in scope. There is a lack of generalized, data-driven frameworks that account for the complex relationships between design parameters, material properties, and performance outcomes specific to demountable connectors. At the same time, machine learning (ML) has proven effective in structural engineering applications, such as predicting material strength, corrosion, and damage progression. ML models can capture nonlinear and multivariable interactions without simplifying assumptions, offering new opportunities for predicting connector behavior. However, their application to demountable systems remains underexplored, largely due to the absence of comprehensive, high-quality datasets.

To bridge this gap, this study develops and compares multiple ML models trained on a hybrid dataset that integrates experimental and numerical data for demountable bolted shear connectors. The objective is to create a reliable predictive tool for estimating ultimate shear capacity, supporting the design of efficient, sustainable, and reusable composite systems.

## Source of dataset

**Fig. 1:**
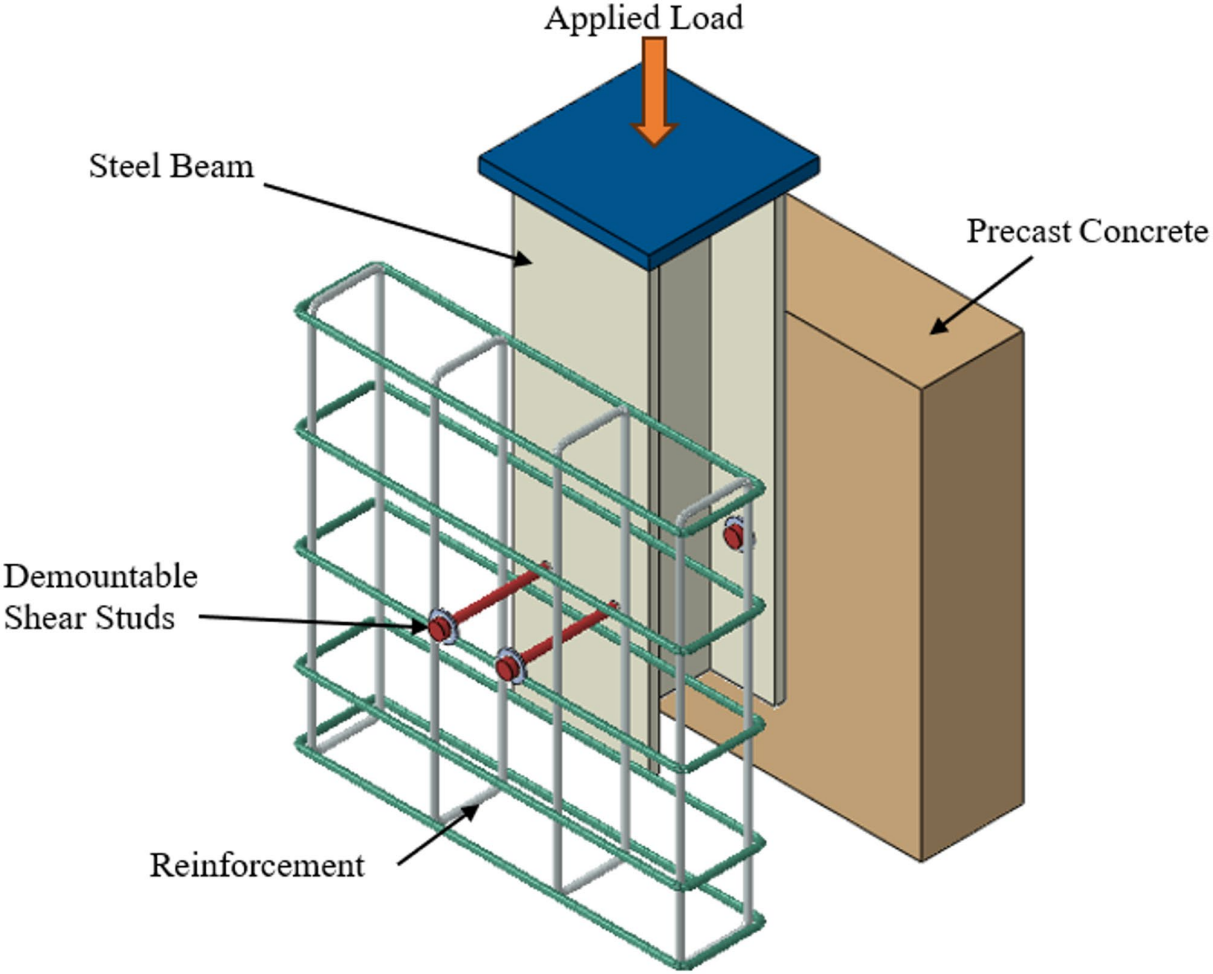
Schematic diagram of the push-out test for demountable shear stud connectors.

**Fig. 2:**
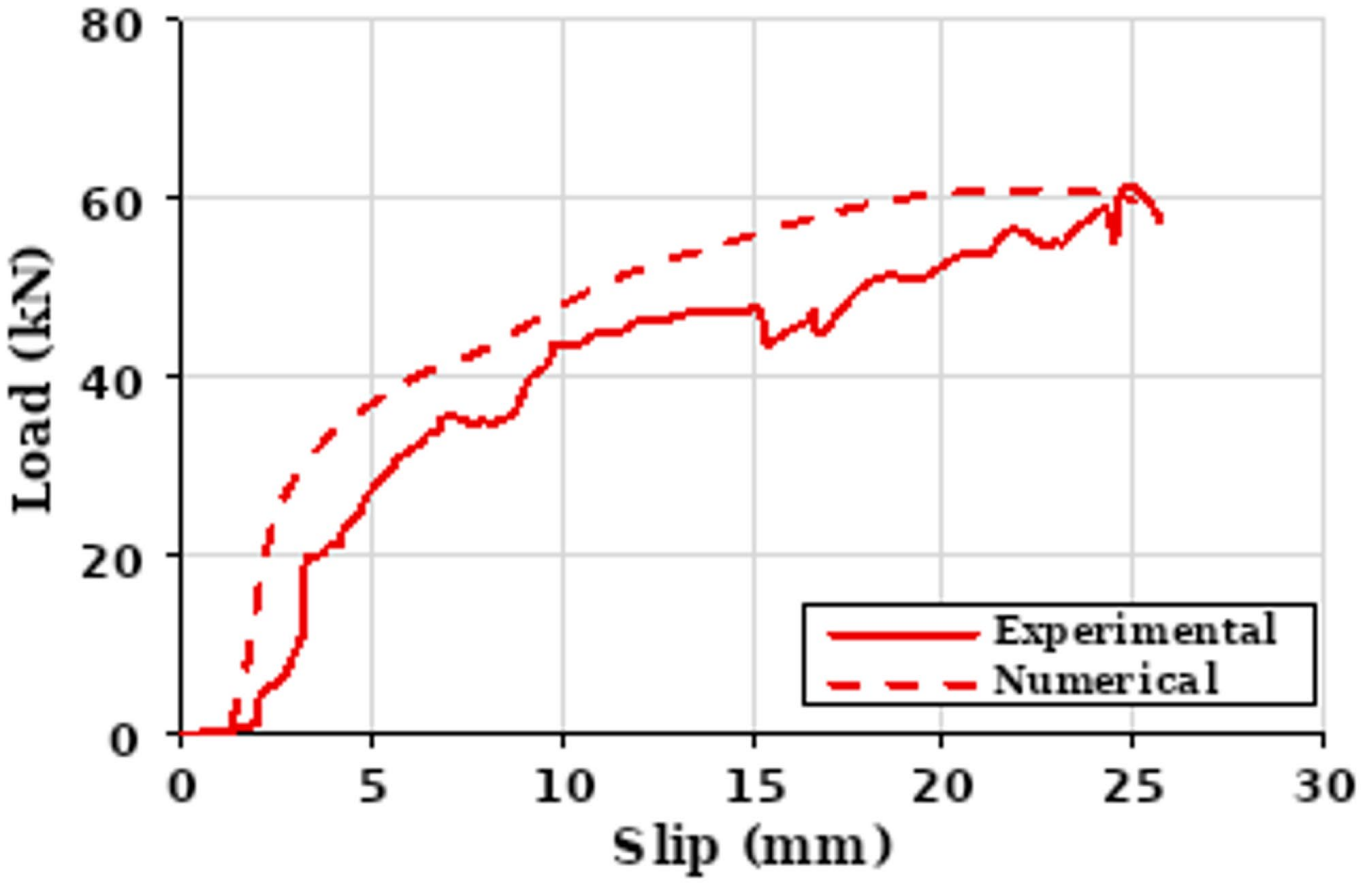
Load–Slip curve for single connector.

The dataset employed in this study was compiled from a combination of experimental results reported in published literature and validated finite element simulations. These sources provided a diverse range of configurations for demountable shear stud connectors as shown in Fig. 1, encompassing variations in geometry, material properties, and structural boundary conditions. Experimental data were extracted from full-scale push-out tests and component-level studies, while simulation data were obtained from nonlinear finite element analyses calibrated against experimental results to ensure accuracy and consistency. A total of 239 experimental and numerical samples were collected, uploaded as a supplementary data. Each capturing a distinct combination of geometric, material, and interface-related parameters relevant to the behavior of demountable shear connectors. The 44.77% experimental and 55.23% numerical data ratio (107 experimental, 132 numerical samples) shapes model training by slightly favoring numerical data’s simulation patterns, potentially limiting the capture of experimental variability, though the latter’s 44.77% share ensures real-world relevance. Normalization mitigates higher experimental variability. Training multiple models with cross-validation leverages the 45:55 split to reduce overfitting, with SHAP plots highlighting feature impacts and confidence intervals confirming robustness, ensuring reliable learning despite numerical dominance as shown in Table 1. To visualize performance and behavior od demountable shear stud connectors. Figure 2 shows a typical load–slip curve based on experimental and simulation data, helping connect the machine learning predictions to real structural behavior. The dataset comprised a mix of input features and a single target output representing the ultimate load capacity Pu of the connector system. Key geometric inputs included bolt diameter and number of bolts, which play a critical role in defining the physical configuration of the connectors. Material properties were also extensively represented, including the compressive strengths of concrete and grout, as well as the yield and ultimate strengths of steel and bolts, reflecting variations in component stiffness and load resistance. To capture interaction behavior at critical connection points, several interfacial friction coefficients such as those between the bolt and concrete, stud and grout, and nut and beam were also included.

**Table 1:** Experimental vs. numerical data distribution.

Data type	Number of samples	Proportion (%)
Experimental	107	44.8
Numerical	132	55.2
Total	239	100

## Selection of input parameters

A total of 13 input parameters were carefully selected to ensure comprehensive representation of the physical and mechanical behaviors that influence the shear capacity of demountable connectors. This selection was guided by both experimental findings and engineering judgment to ensure that the chosen features reflect the key factors governing load transfer in steel–concrete composite systems. Together, these parameters enable the development of robust and accurate machine learning models capable of capturing the complex interactions within the connector system. The mechanical properties include the compressive strength of concrete *F*_*cu, C*_ which defines the load-bearing capacity of the surrounding matrix, and the grout compressive strength *F*_*cu, G*_, which plays a role in confinement and localized stress distribution. The stud connector diameter Db is a primary geometric parameter due to its direct impact on the shear-resisting area. Additionally, the yield strength *F*_*y, b*_ and ultimate tensile strength *F*_*u, b*_ of the connector describes its material behavior under load, while the applied pretension force Tb affects both the initial stiffness and the slip characteristics at the interface. Recognizing the importance of interfacial behavior in composite action, the dataset al.so includes friction coefficients at seven key contact interfaces: beam–concrete, stud–concrete, grout–concrete, stud–grout, stud–beam, grout–beam, and nut–beam (these friction coefficients were not considered in previous studies)^33,36,37,39^. These parameters help capture the resistance to relative movement between materials and allow the models to consider interaction effects that significantly influence shear transfer. By integrating both material and interface-related features, the selected input set provides a holistic foundation for accurately predicting the performance of demountable shear connectors under load.

## Data normalization and splitting

Before training the machine learning models, the data needed to be adjusted to a consistent scale so that all features could be fairly considered during the learning process. Since the dataset included 13 input parameters with different measurement units and value ranges similar bolt diameter, compressive strength, and friction coefficients normalizing the data helped prevent any one feature from overpowering the others. To do this, the values in each feature were scaled between 0 and 1 by dividing each value by the feature’s maximum using MinMaxScaler from scikit-learnc. Once normalized, the 239 data records were shuffled to remove any order-related bias and then split into two sets: 70% of the data was used to train the models, while the remaining 30% was set aside to test how well the models could predict new, unseen results. This approach helped ensure that the models were both accurate and reliable.

## Overview of machine learning algorithms

1. Linear regression is a foundational supervised learning method that models the relationship between a continuous target variable and one or more input features^40^. It assumes a linear association between inputs and output defined in Eq. (1), making it suitable for problems with simple dependencies. While it is computationally efficient and easy to interpret, its limitations become evident in datasets with complex, nonlinear structures or multicollinearity.
  1 $$\:y = \beta \:_{0} + \beta \:_{1} x + \varepsilon$$
  where $\:{\beta\:}_{0}$​ is the intercept, $\:{\beta\:}_{1}$​ is the slope, and $\varepsilon$ is the error term. This method is straightforward and understandable, making it appropriate for datasets with linear relationships between variables.
2. Ridge regression is an extension of linear regression that incorporates L2 regularization. By penalizing large coefficient values, it addresses multicollinearity among features and enhances the model’s stability. Ridge regression retains all features in the model, making it useful when all input variables are believed to contribute to the outcome as shown in Eq. (2), though it may struggle with variable selection^34^.
  2 $$\:y={\beta\:}_{0}+{\beta\:}_{1}x+\lambda\:\sum\:{\beta\:}^{2}$$
  where $\:\lambda\:$ is the regularization parameter.
3. Lasso regression introduces L1 regularization to the linear regression framework^41^. This regularization technique not only reduces overfitting but also enables automatic feature selection by shrinking less important feature coefficients to zero as defined in Eq. (3). It is particularly advantageous in high-dimensional datasets where only a subset of features is expected to influence the target.
  3 $$\:y={\beta\:}_{0}+{\beta\:}_{1}x+\lambda\:\sum\:\left|\beta\:\right|$$
4. The decision tree regressor is a non-parametric model that predicts output by learning simple decision rules inferred from the input data^41^. The model recursively partitions the dataset based on feature thresholds, forming a tree structure where each branch represents a decision rule. This approach effectively captures nonlinearity and feature interactions, but it may overfit unless pruning or depth constraints are applied.
5. Random Forest is an ensemble method that combines the outputs of multiple decision trees to produce a robust prediction. Each tree is trained on a random subset of data and features, ensuring diversity among trees. The model mitigates overfitting and improves generalization by averaging the results from all trees. It is widely used in engineering and science for its accuracy and ability to handle high-dimensional and noisy data^41^.
6. Support Vector Regression is an adaptation of the support vector machine algorithm for regression tasks. It aims to find a function that approximates the target values within a predefined margin while maintaining the flattest possible prediction curve. SVR uses kernel functions to model nonlinear relationships and is known for its robustness and ability to generalize well in cases of limited or high-dimensional data^42^.
7. The K-Nearest Neighbors Regressor is a simple, instance-based learning algorithm that makes predictions by averaging the outputs of the closest training samples to a new input^43^. It does not require training in the traditional sense and relies heavily on the local structure of the data. Its effectiveness depends on the choice of distance metric and the number of neighbors considered.
8. XGBoost is a highly efficient and scalable implementation of gradient boosting that builds models sequentially by correcting the errors of previous models^44^. It incorporates regularization techniques to control model complexity and prevent overfitting. XGBoost is well-regarded for its performance in regression and classification tasks across various domains, including structural engineering and data science.

## Evaluation metrics

To assess the predictive performance of the developed machine learning models, several commonly used regression evaluation metrics were employed: the coefficient of determination (*R²*) Eq. (4), Root Mean Square Error (RMSE) Eq. (5), and Mean Absolute Error (MAE) Eq. (6). These metrics provide a comprehensive understanding of model accuracy, precision, and generalization capability.

The *R²* measures the proportion of variance in the target variable that is predictable from the input features. A value closer to 1 indicates a strong correlation between the predicted and actual values, reflecting a high-quality fit. RMSE, on the other hand, quantifies the average magnitude of prediction error, giving more weight to larger errors. It is particularly useful in structural applications where large deviations can be critical. MAE represents the average of the absolute differences between predicted and actual values, offering a straightforward interpretation of model accuracy that is less sensitive to outliers compared to RMSE.

4 $$\:{R}^{2}=1-\frac{\sum\:{\left({y}_{i}-{\widehat{y}}_{i}\right)}^{2}}{\sum\:{\left({y}_{i}-\stackrel{-}{y}\right)}^{2}}$$

5 $$\:MSE=\frac{1}{n}\sum\:_{i=1}^{n}{\left({y}_{i}-{\widehat{y}}_{i}\right)}^{2}$$

6 $$\:MAE=\frac{1}{n}\sum\:_{i=1}^{n}\left|{y}_{i}-{\widehat{y}}_{i}\right|$$

where: $\:{y}_{i}$ are the observed values, $\:{\widehat{y}}_{i}$ are the predicted values, $\:\stackrel{-}{y}$ is the mean of the observed values.

## Statistical description

**Fig. 3:**
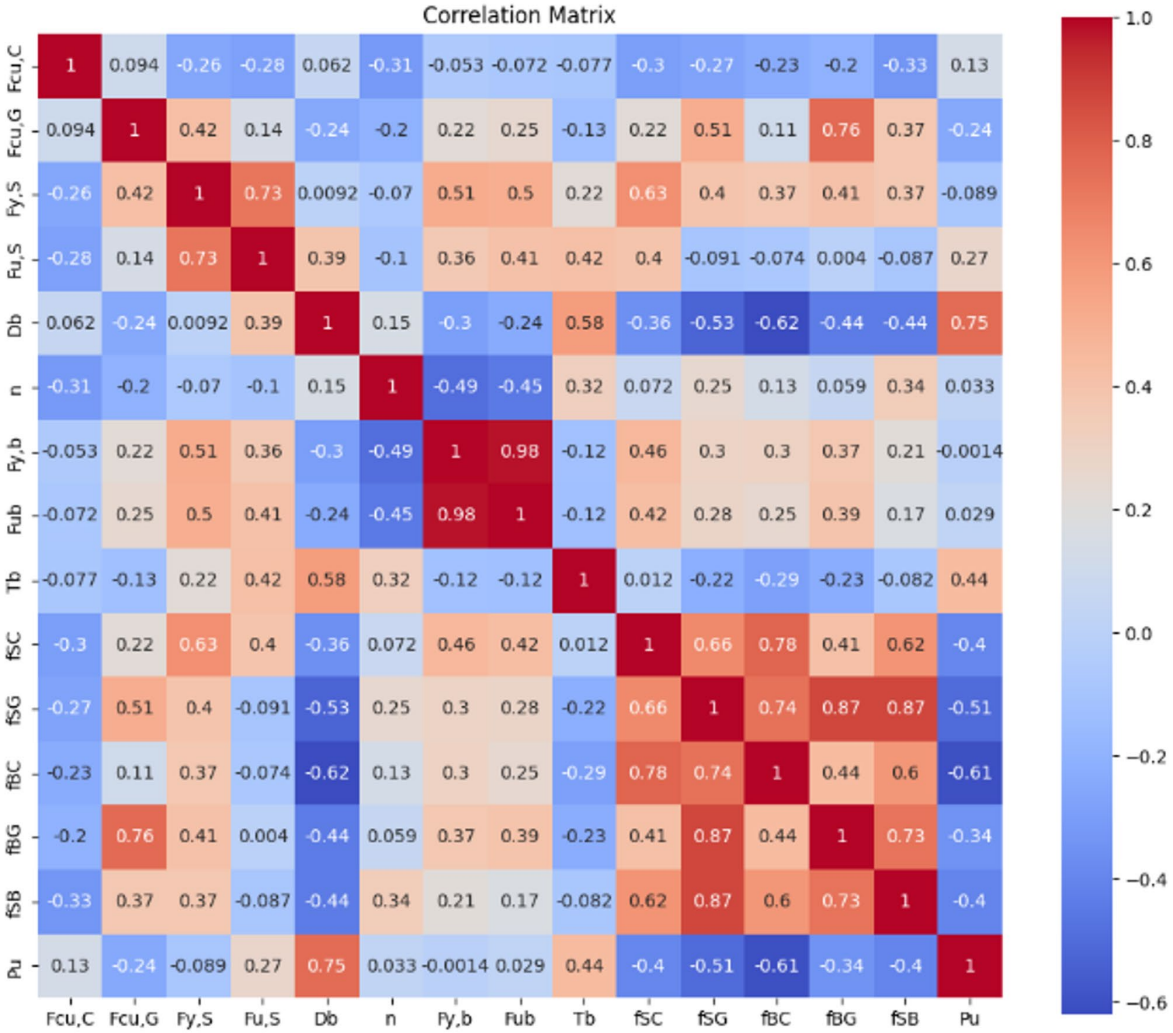
Pearson correlation among features.

A Pearson correlation analysis was carried out to examine the linear relationships between the selected input parameters and the ultimate shear load, as illustrated in Fig. 3. The results reveal that the bolt diameter *D*_*b*_ exhibits the strongest positive correlation with the load (*r* = 0.75), confirming its critical role in increasing the effective shear area and mechanical interlock. Additionally, the stud connector pretension force Tb shows a moderate positive correlation with load (*r* = 0.44), indicating that higher axial clamping contributes to improved load transfer and overall system resistance.

In contrast, several friction-related parameters demonstrate moderate to strong negative correlations with the load. Notably, the friction between bolt and concrete *f*_*BC*_, stud and grout *f*_*SG*_, and stud and beam *f*_*SB*_ show negative relationships of *r* = − 0.61, − 0.51, and − 0.40, respectively. These results suggest that excessive friction at these interfaces may lead to early relative movement or reduced energy dissipation, thereby affecting the effective shear transfer mechanisms.

Material strength parameters such as grout compressive strength *F*_*cu, G*_, concrete compressive strength *F*_*cu, C*_, and steel yield strength *F*_*y, S*_ show relatively weak correlations with the load, ranging between *r* = − 0.24 and 0.13. This indicates that while these variables are necessary for defining material behavior, they may not be the primary drivers of shear resistance in demountable connections, where interface behavior and geometry play larger roles.

A particularly strong correlation is observed between bolt yield strength *F*_*y, b*_ and ultimate strength F_ub_, with a near-perfect value of *r* = 0.98, reflecting their expected consistency in standardized bolt materials. Additionally, multicollinearity is evident among several friction parameters, particularly between *f*_*SC*_, *f*_*SG*_, and *f*_*BG*_, where correlation values exceed *r* = 0.85. These interdependencies should be addressed in the modeling process through dimensionality reduction or regularization to improve model stability and interpretability.

Table 2 offers a clear picture of how varied and well-distributed the dataset is, which plays a key role in building effective machine learning models. For example, bolt diameter *D*_*b*_ ranges from 6 *mm* to 27 *mm*, and the number of bolts *N* varies between 4 and 8. Both features show a healthy amount of variation, which allows the model to learn how different sizes and quantities of connectors impact shear performance. The dataset also includes a broad range of bolt yield *F*_*y, b*_ and ultimate strength *F*_*ub*_, with values stretching from around 250 MPa up to over 1300 MPa. This wide spread helps the model capture how differences in material strength affect the connectors’ behavior under load.

**Table 2:** Descriptive statistics of the numerical features.

Parameter	Mean	STD	Min.	25%	50%	75%	Max.
Input							
F_cu, C_ (Mpa)	37.59707	12.26444	23.06	30	30	48.2	65
F_cu, G_ (Mpa)	14.4749	26.68559	0	0	0	31.62	95.1
F_y, S_ (Mpa)	325.9478	39.95516	235	280.3	342	355	375
F_u, S_ (Mpa)	447.6119	62.32162	350	387.5	470	479.2	541
D_b_(mm)	16.30962	4.480156	6	12	16	20	27
N	5.389121	1.90842	4	4	4	8	8
F_y, b_ (Mpa)	656.3626	266.178	248.81	330	800	830	1115
F_ub_ (Mpa)	817.6174	285.3866	385.8	540	955	1035	1319
T_b_ (kN)	48.51326	42.99424	0	20	27	95	155
f_SC_	0.293431	0.1749	0.1	0.1	0.3	0.45	0.7
f_SG_	0.090377	0.204856	0	0	0	0	0.7
f_BC_	0.277908	0.156661	0	0.25	0.25	0.3	0.7
f_BG_	0.138703	0.267021	0	0	0	0	0.7
f_SB_	0.302092	0.132747	0	0.25	0.25	0.3	0.7
Output							
P_u_ (kN)	122.3347	67.2099	5.5	68.5	112.5	183.75	347.5

Even though some friction-related features like *f*_*SC*_, *f*_*SG*_, and *f*_*BC*_ have lower average values, they still cover a wide range (from 0 to 0.7), making them important for understanding the role of interface behavior in load transfer. The target variable, ultimate load capacity *P*_*u*_, also shows significant variability from as low as 5.5 kN up to 347.5 kN which means the dataset captures both weak and strong connection performances. This kind of diversity in the data helps ensure that the machine learning models can learn from a wide range of scenarios and make accurate, generalizable predictions.

## Prediction performance of machine learning models

**Fig. 4:**
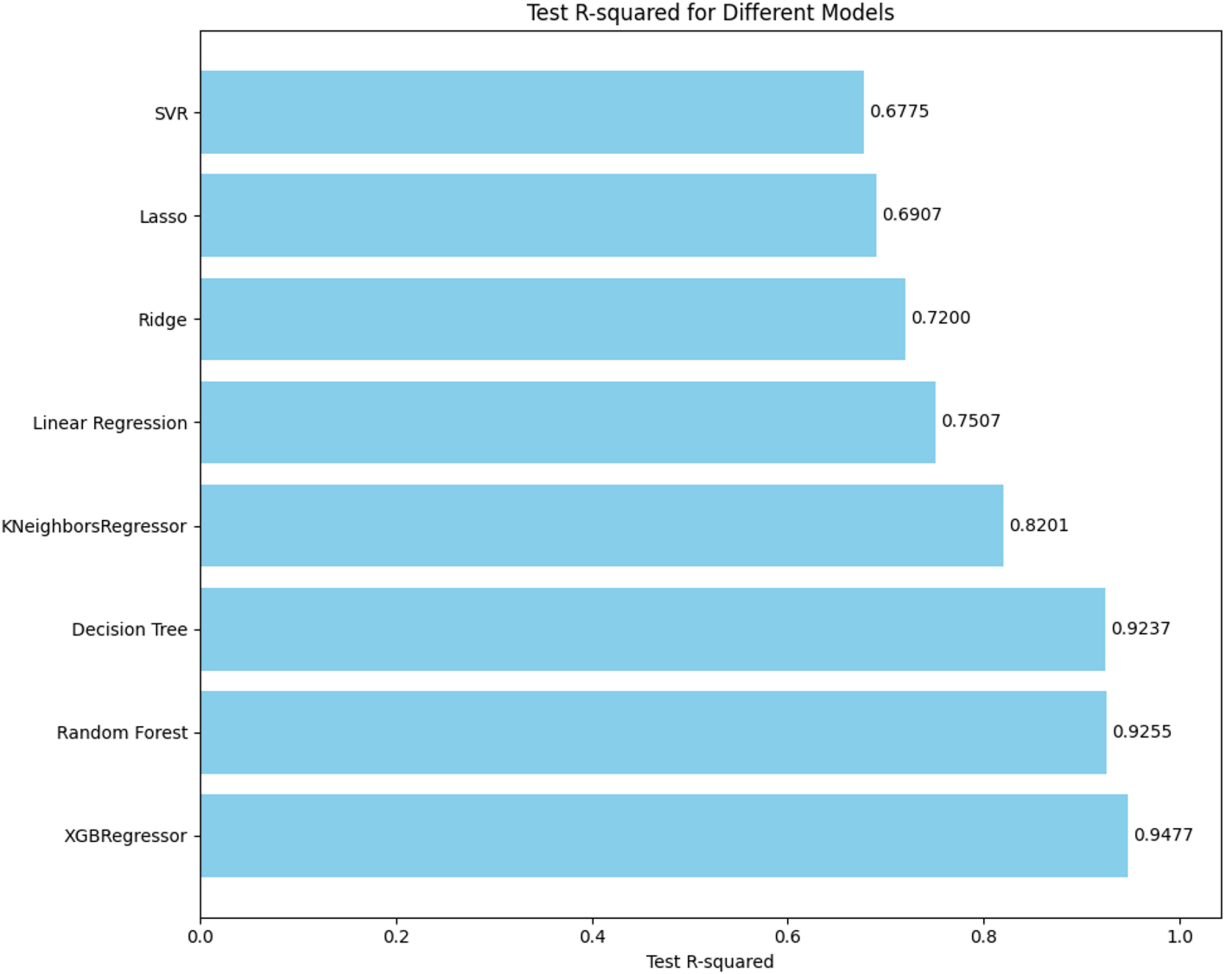
Tested R^2^ for different models.

The bar chart in Fig. 4 presents the R-squared (*R²*) scores for various machine learning regression models applied to predict the ultimate load capacity of demountable shear connectors. Among all the tested models, XGBoost Regressor delivered the best performance, achieving an impressive *R²* value of 0.9477, indicating that nearly 95% of the variability in the output could be explained by the model. Close behind, Random Forest achieved an *R²* of 0.9255, also demonstrating excellent predictive capability. The strong performance of these ensemble-based models can be attributed to their ability to handle nonlinear relationships and complex feature interactions through iterative learning and model averaging.

The Decision Tree Regressor also performed well, with an *R²* score of 0.9237, showing that even a single-tree structure can effectively capture patterns in the data when nonlinearity is present. The K-Nearest Neighbors (KNN) Regressor followed with a moderate *R²* value of 0.8201, suggesting that similarity-based methods can be effective with appropriate tuning, though they may lack the flexibility of ensemble approaches.

In contrast, linear models such as Linear Regression (*R²* = 0.7507), Ridge (*R²* = 0.7200), and Lasso (*R²* = 0.6907) showed lower performance, indicating that their assumption of linearity limits their ability to fully capture the complexity of the dataset. Finally, Support Vector Regression (SVR) produced the lowest *R²* value at 0.6775, reflecting reduced predictive accuracy. This underperformance could be due to sensitivity in kernel selection and poor generalization in high-dimensional feature spaces. These findings highlight that ensemble tree-based models, particularly XGBoost and Random Forest, are the most reliable for modeling complex structural behaviors in demountable shear connector systems.

**Fig. 5:**
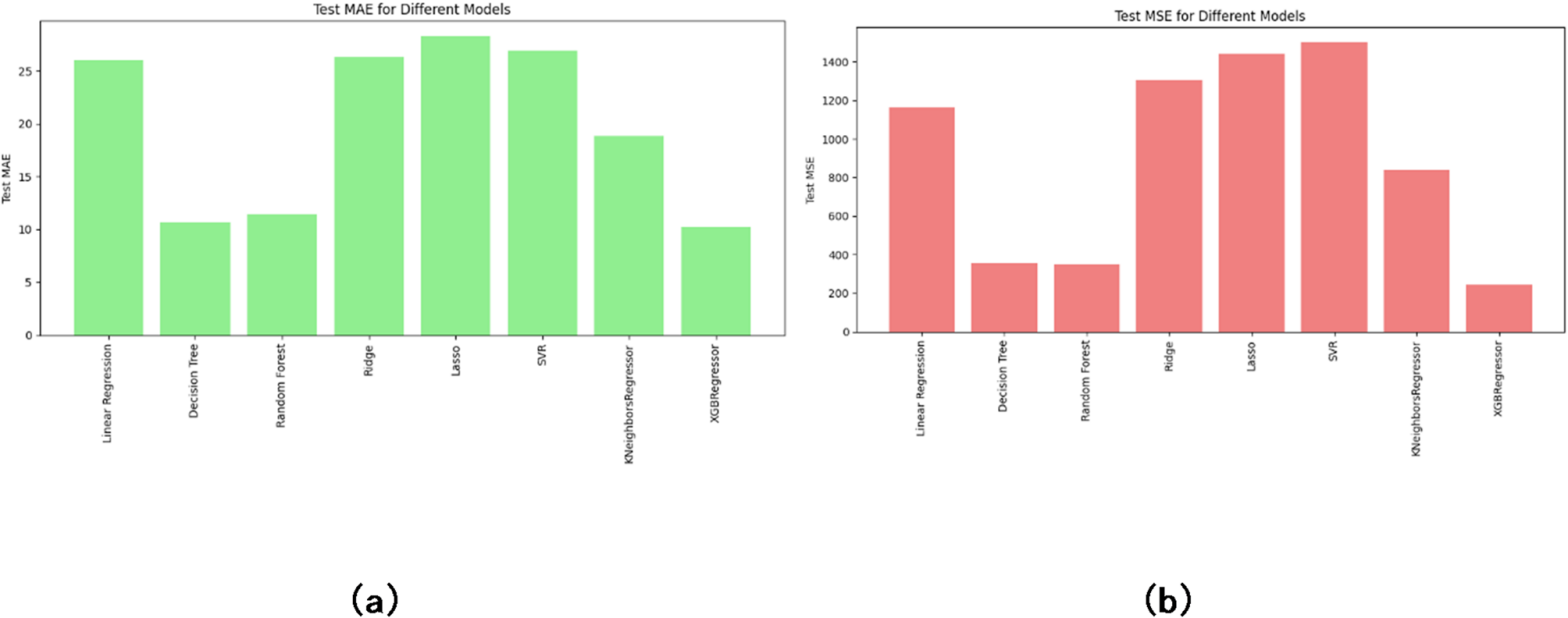
Bar charts for (**a**) Mean Absolute Error. (**b**) Mean Squared Error.

Figure 5(a) presents the Mean Absolute Error (MAE) for each regression model used in predicting the shear load of demountable shear connectors. MAE measures the average difference between predicted and actual values, offering a straightforward interpretation of accuracy. From the results, the XGBoost Regressor stands out with the lowest MAE, around 10, indicating that its predictions were consistently close to the true values. Random Forest and Decision Tree also performed well, with MAE values only slightly higher, showing their reliability in minimizing average prediction error. In contrast, Lasso, Ridge, and Linear Regression models reported the highest MAE values (above 25), reflecting less accurate and more variable predictions. SVR and K-Nearest Neighbors fell in the middle, performing better than linear models but not quite reaching the precision of ensemble methods. These results reinforce that tree-based and boosting algorithms offer better consistency in prediction when dealing with nonlinear structural behavior.

Figure 5(b) shows the Mean Squared Error (MSE) values for the same models, which penalize larger errors more heavily and provide a clearer picture of model robustness. Once again, XGBoost achieved the best performance, with an MSE of approximately 300, indicating both accuracy and resilience to large prediction errors. Random Forest and Decision Tree followed closely, with relatively low MSE values that reflect their strength in handling complex feature interactions. On the other hand, Lasso and Ridge Regression recorded the highest MSE scores (around 1400), suggesting that these models are more prone to large deviations between predicted and actual values. Linear Regression and SVR also showed weaker performance, with elevated MSE values compared to the top-performing models. K-Nearest Neighbors, while not the most precise, showed moderate MSE, positioning it as a viable option under certain conditions. Overall, the MSE results further confirm that XGBoost and Random Forest are the most robust models, making them strong candidates for practical engineering applications where predictive accuracy is essential.

Table 3 presents 95% confidence intervals (CI) for R², Mean Squared Error (MSE), and Mean Absolute Error (MAE) for various regression models evaluated on a dataset. The XGBRegressor and Decision Tree models exhibit the highest R² CI ranges (0.8958–0.9759 and 0.8440–0.9758, respectively), indicating strong predictive performance. In contrast, Lasso and SVR have the lowest R² CI ranges (0.5554–0.7860 and 0.5615–0.8016) and wider MSE CIs (922.90-2057.40 and 784.08-2375.08), reflecting poorer fit and higher variability.

**Table 3:** Confidence intervals for all models.

Model name	*R*^2^ CI (95%)	MSE CI (95%)	MAE CI (95%)
Linear regression	0.6474–0.8226	775.08-1622.89	20.99–31.45
Decision tree	0.8440–0.9758	113.24-659.81	7.19–14.66
Random forest	0.8288–0.9696	139.47-699.13	8.46–15.27
Ridge	0.6147–0.8073	816.65-1879.35	20.99–31.72
Lasso	0.5554–0.7860	922.90-2057.40	22.86–34.23
SVR	0.5615–0.8016	784.08-2375.08	20.58–33.38
KNeighborsRegressor	0.7036–0.9049	397.67-1420.08	13.81–24.54
XGBRegressor	0.8958–0.9759	126.60-430.61	7.94–13.20

## Machine learning models versus experimental results

**Fig. 6:**
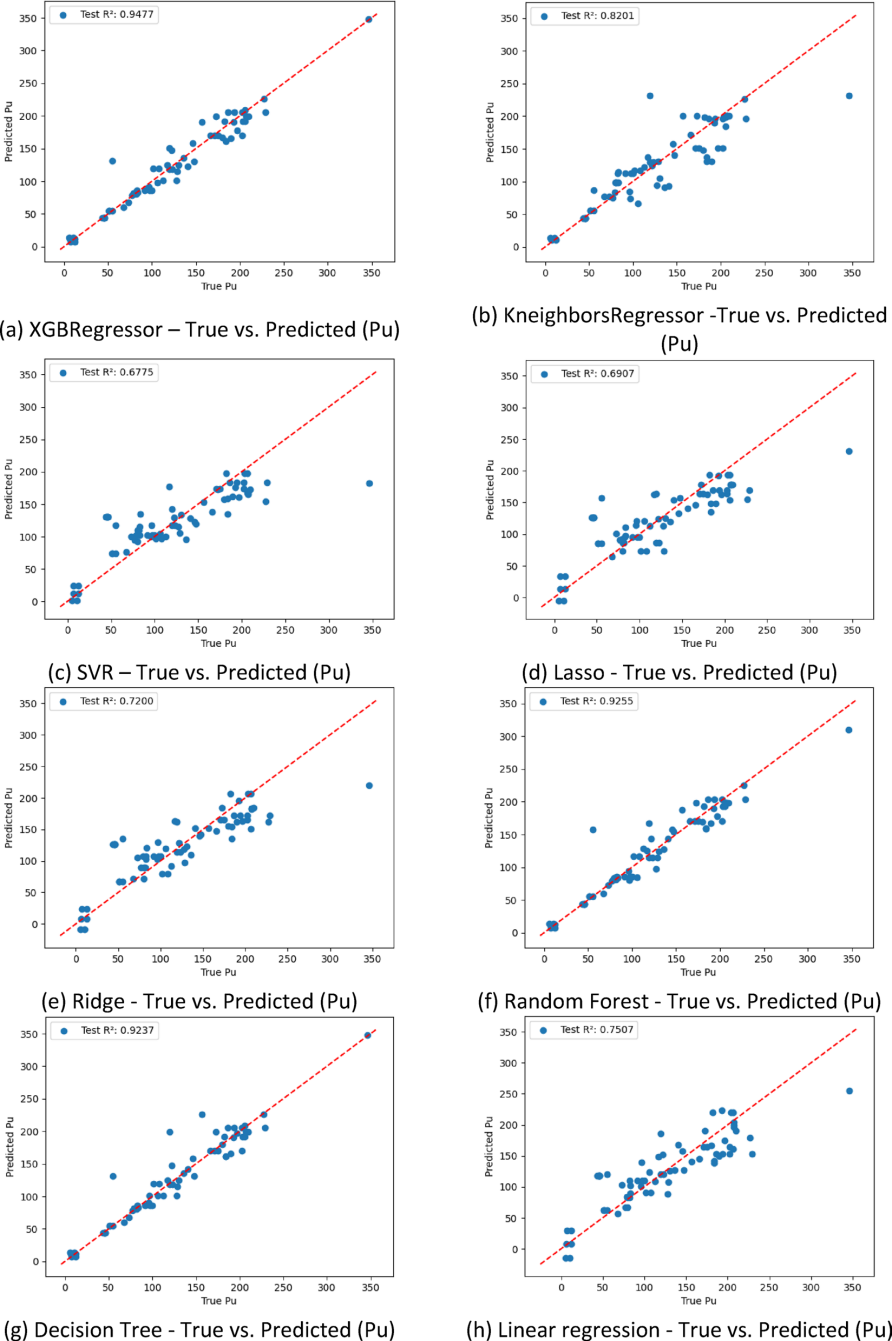
Predicted shear resistance from ML models versus experimental results.

Figure 6 shows the scatter plots of predicted versus actual shear load Pu for eight different machine learning models, providing a visual assessment of each model’s predictive performance. The ideal outcome is represented by the red dashed line, where predicted values perfectly match the true experimental results.

Among all the models, XGBoost Fig. 6(a) achieved the best performance with an *R²* value of 0.9477, closely followed by Random Forest Fig. 6(f) and Decision Tree Fig. 6(g), with *R²* values of 0.9255 and 0.9237, respectively. These ensemble and tree-based methods demonstrated excellent alignment with the experimental data, confirming their ability to accurately capture nonlinear relationships in the dataset. Predictions using these models clustered tightly around the ideal line, indicating both precision and consistency.

The K-Nearest Neighbors model Fig. 6 (b) also delivered acceptable performance (*R²* = 0.8201), though with slightly greater dispersion, especially at higher load values. Linear Regression Fig. 6 (h), Ridge Fig. 6 (e), and Lasso Fig. 6 (d) showed moderate performance, with *R²* values of 0.7507, 0.7200, and 0.6907, respectively. While they captured general trends in the data, their predictive accuracy decreased as the complexity of the relationships increased, which is expected given their linear nature.

On the other end of the spectrum, Support Vector Regression Fig. 5(c) exhibited the weakest performance, with an *R²* of just 0.6775. The wider scatter and deviation from the ideal line suggest a limited ability to model the nonlinear and high-dimensional behavior characteristic of shear connector performance.

**Fig. 7:**
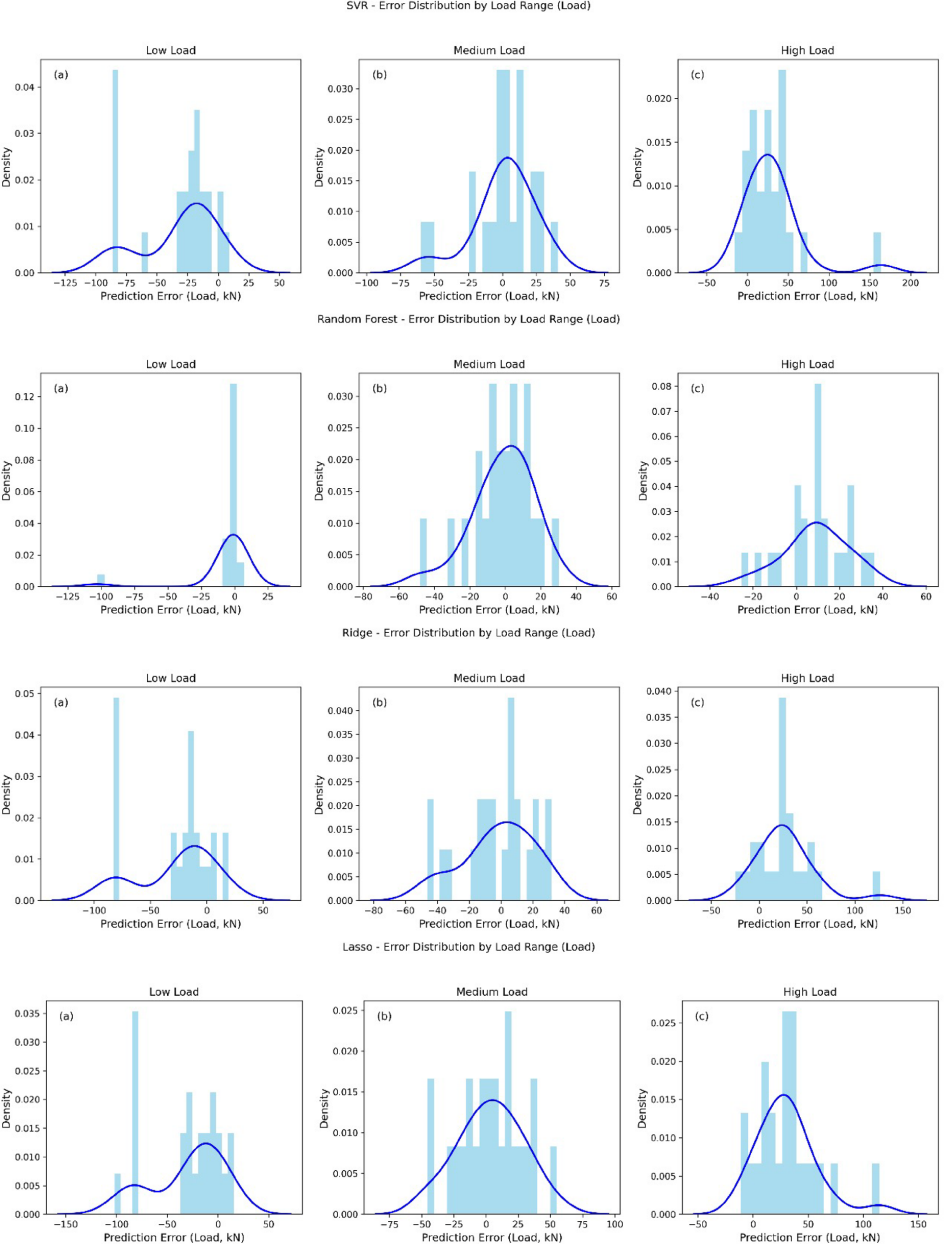

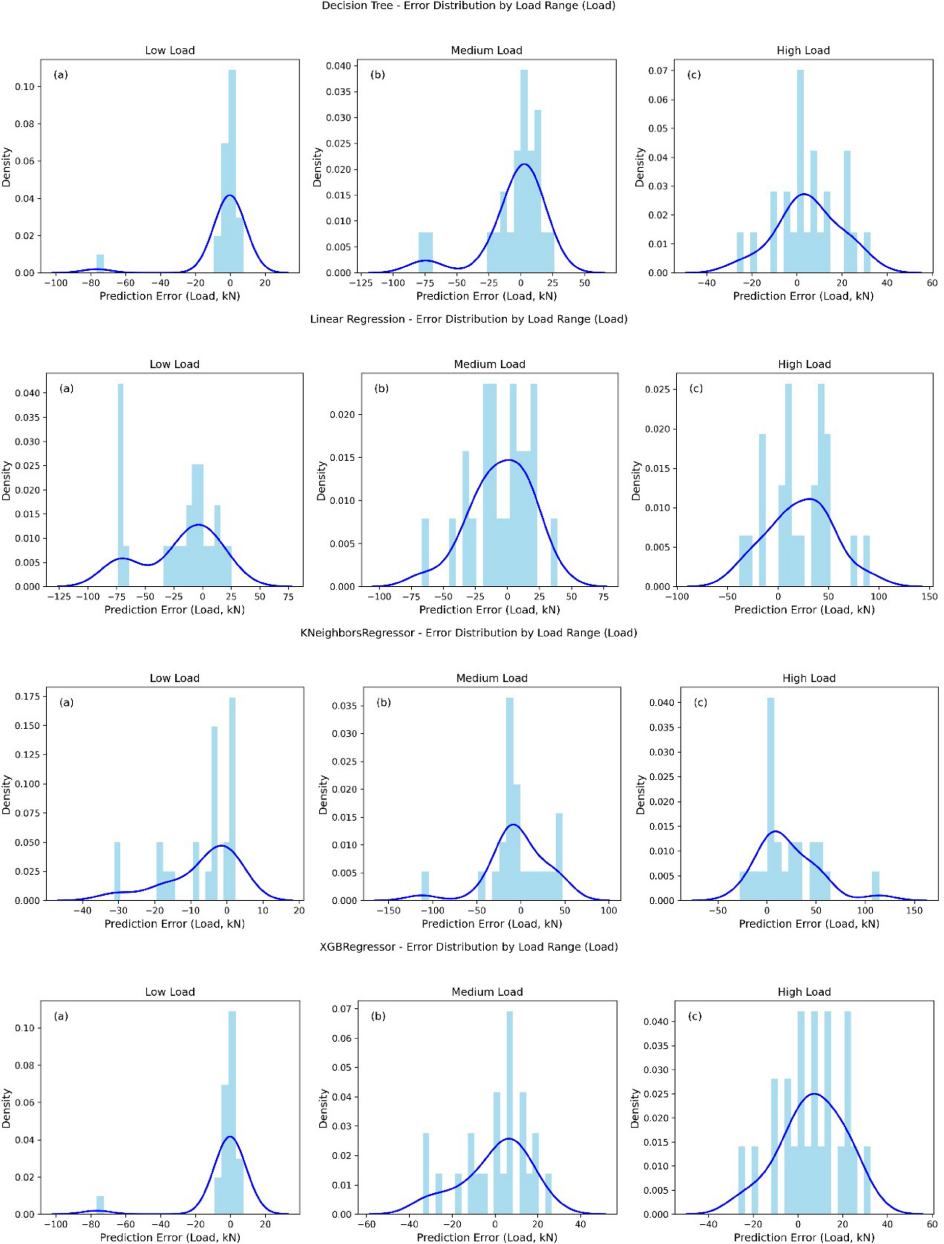
Error histograms for all models across different load ranges.

The error distribution plots for Decision Tree, XGBoost Regressor, KNeighborsRegressor, Lasso, Linear Regression, Random Forest, Ridge, and SVR across low, medium, and high load ranges provide a comprehensive view of prediction accuracy for each model as shown in Fig. 7. For low load, all models exhibit a pronounced peak in error distribution around 0 kN, with a long tail towards negative errors (e.g., Decision Tree and XGBoost show errors up to -100 kN), indicating a tendency to underestimate load, though the density is highest near zero. In the medium load range, most models (e.g., XGBoost, Random Forest, Ridge, SVR) display a more symmetric error distribution centered around 0 kN with a bell-shaped curve, suggesting improved accuracy and balanced over- and underestimation, though outliers persist (e.g., Lasso and Linear Regression show errors up to -100 kN). For high load, the error distributions shift towards positive values (e.g., 0 to 60 kN for Decision Tree and Random Forest), reflecting a tendency to overestimate, with SVR and XGBoost showing the densest peaks around 0 to 50 kN, indicating better precision in this range. Across all models, the error variance increases with load range, with high load predictions showing the widest spread (e.g., up to 200 kN for SVR), suggesting greater uncertainty.

**Fig. 8:**
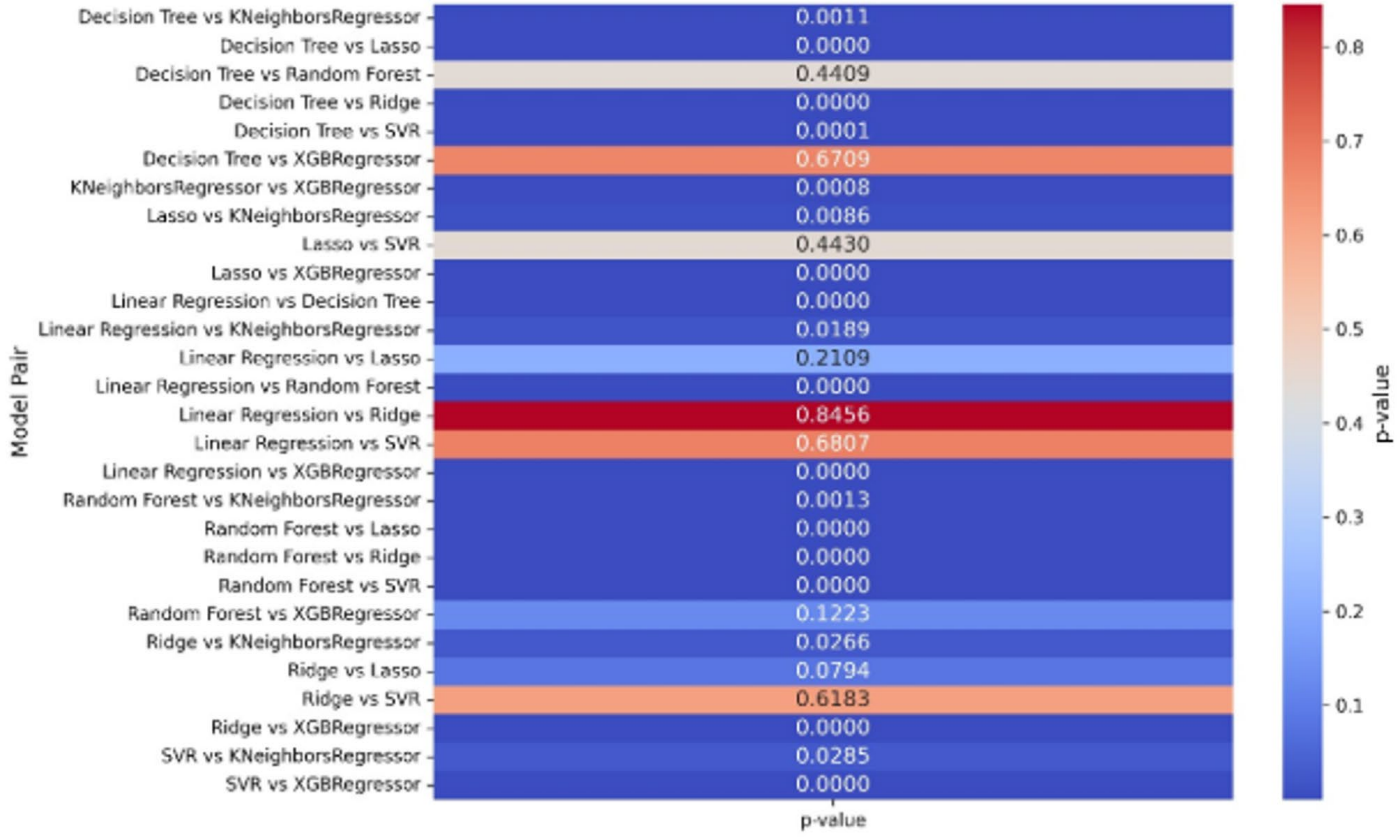
Paired t-test.

Significant variations in predictive accuracy are shown by the heatmap that compares the p-values from paired t-test between the models (Decision Tree, KNeighborsRegressor, Lasso, Linear Regression, Random Forest, Ridge, SVR, and XGBoostRegressor) are shown in Fig. 8. Comparisons like Decision Tree vs. Lasso, Linear Regression vs. Random Forest (0.8456), and Ridge vs. SVR (0.6183) show notable significant p-values (e.g., < 0.001), showing different error distributions or prediction accuracies between these models. Blue hues (e.g., Lasso vs. XGBoostRegressor at 0.0000) show highly significant disparities, whereas strong red hues (e.g., Decision Tree vs. XGBoostRegressor at 0.6709) reflect less significant variances, signaling similar performance. Ridge and SVR show moderate differences (e.g., 0.0266 and 0.0285), but models like as Random Forest and XGBoostRegressor consistently show significance against others (e.g., *p* < 0.001), indicating higher robustness.

## SHAP-based explanation of ML models

**Fig. 9:**
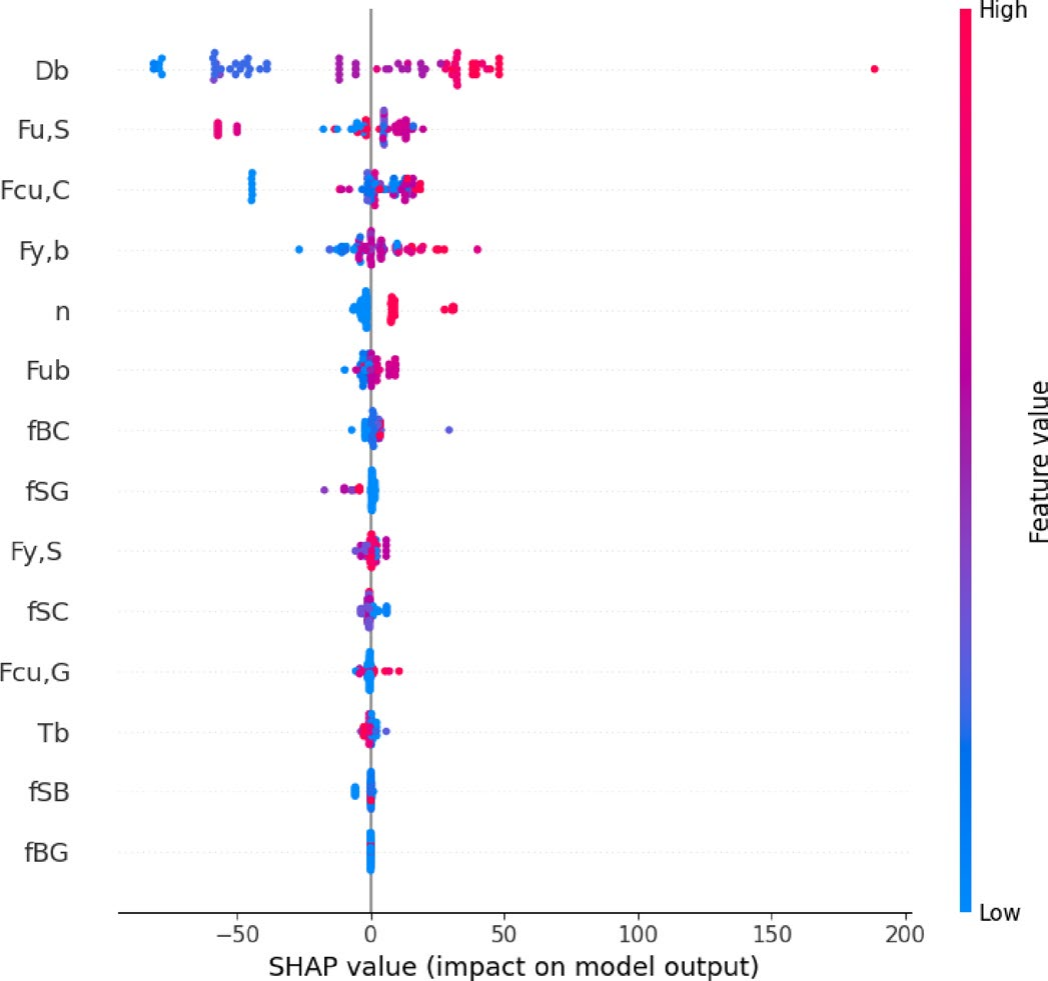
SHAP summary plot.

Figure 9 presents a SHAP summary plot, which offers a transparent and model-agnostic interpretation of how each feature influences the predicted ultimate load *P*_*u*_ in the machine learning model. Each dot in the plot represents a single prediction, with its horizontal position showing the feature’s impact on the model output, while the color gradient reflects the value of the feature from low (blue) to high (red).

From the plot, it is clear that bolt diameter *D*_*b*_ has the most significant impact on the predicted load, with higher values consistently pushing predictions upward. This reaffirms its physical relevance, as larger diameters naturally contribute to greater shear resistance. Other important features include the ultimate strength of steel *F*_*u, S*_, compressive strength of concrete *F*_*cu, C*_, and bolt yield strength *F*_*y, b*_, all of which show considerable influence on the model predictions. These material properties play a direct role in determining how the connector performs under load.

Additionally, the number of bolts *N* and ultimate strength of bolts *F*_*ub*_ contribute positively to model output, indicating that increasing either the connector counts or material capacity enhances the predicted shear load. Conversely, several interface-related friction coefficients, such as *f*_*BC*_ (bolt–concrete), *f*_*SG*_ (stud–grout), and *f*_*SC*_ (stud–concrete), show more scattered and subtle effects, sometimes positively influencing the output and other times reducing it depending on their value and interaction context.

Less influential features, including grout compressive strength *F*_*cu, G*_, pretension force *T*_*b*_, and friction at beam–grout *f*_*BG*_ or stud–beam *f*_*SB*_ interfaces, exhibit minimal impact, clustering closely around zero on the SHAP value axis. This suggests that although these variables are part of the connection system, they contribute less to the model’s decision-making process in predicting shear resistance.

## Feature importance

**Fig. 10:**
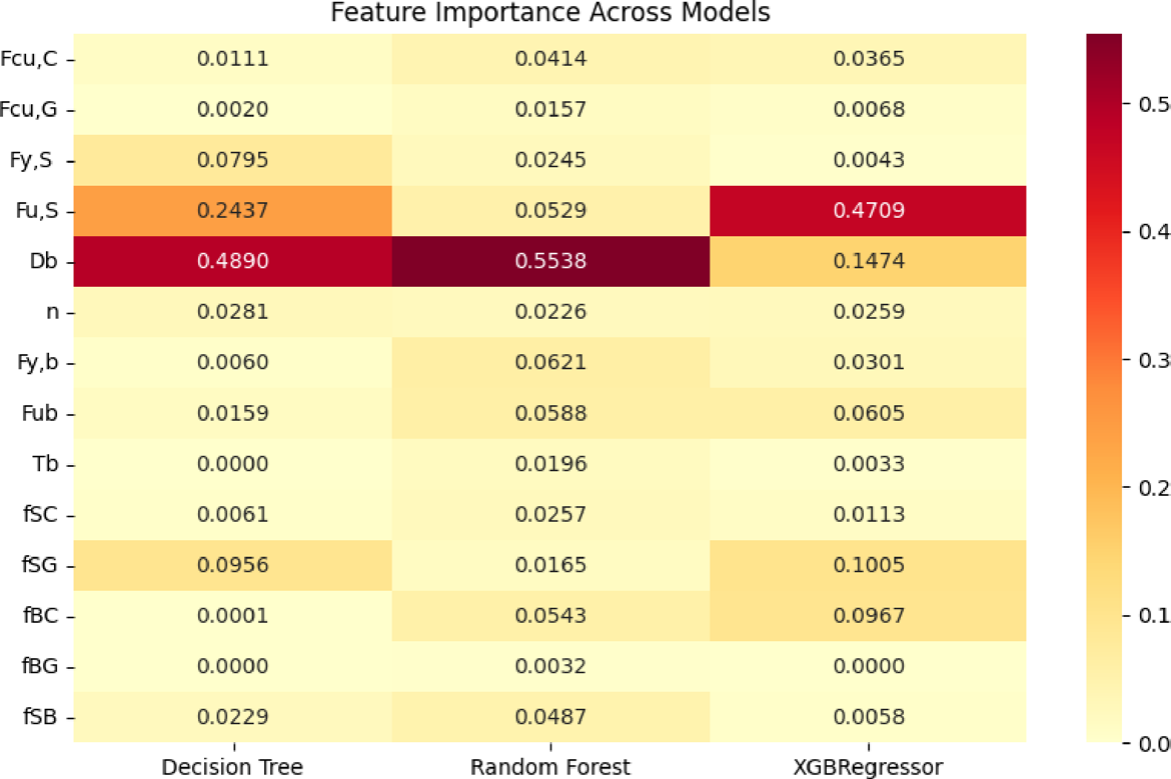
Impact of each feature on shear capacity of single stud.

The final shear capacity of demountable shear connectors was predicted using three machine learning models: Decision Tree, Random Forest, and XGBoost Regressor. A comparative heatmap of feature importance scores for each model is presented in Fig. 10, where darker shades indicate higher influence, and the numeric values reflect the relative contribution of each input to the model’s predictive performance. Bolt diameter *D*_*b*_ consistently emerged as the most dominant feature across all models, with the highest importance in Random Forest (0.5538) and Decision Tree (0.4890), highlighting the critical role of connector geometry in determining load-bearing behavior.

In the XGBoost model, steel ultimate strength *F*_*u, S*_ had the most pronounced influence (0.4709), indicating that material strength plays a significant role in shaping the model’s predictions in gradient boosting frameworks. Additionally, interface-related parameters, particularly stud–grout friction *f*_*SG*_ and bolt–concrete friction *f*_*BC*_, showed notable importance in XGBoost, with scores of 0.1005 and 0.0967, respectively. These values reinforce the relevance of local interface behavior in accurately modeling shear resistance.

Other features, such as the number of bolts, bolt yield strength, and bolt ultimate strength, had moderate influence, while parameters like grout strength *F*_*cu, G*_, steel yield strength *F*_*y, S*_, and several interface friction terms contributed marginally. The results confirm that geometric and material properties are the primary drivers of shear capacity prediction, with interface effects contributing more prominently in complex, non-linear models like XGBoost.

**Fig. 11:**
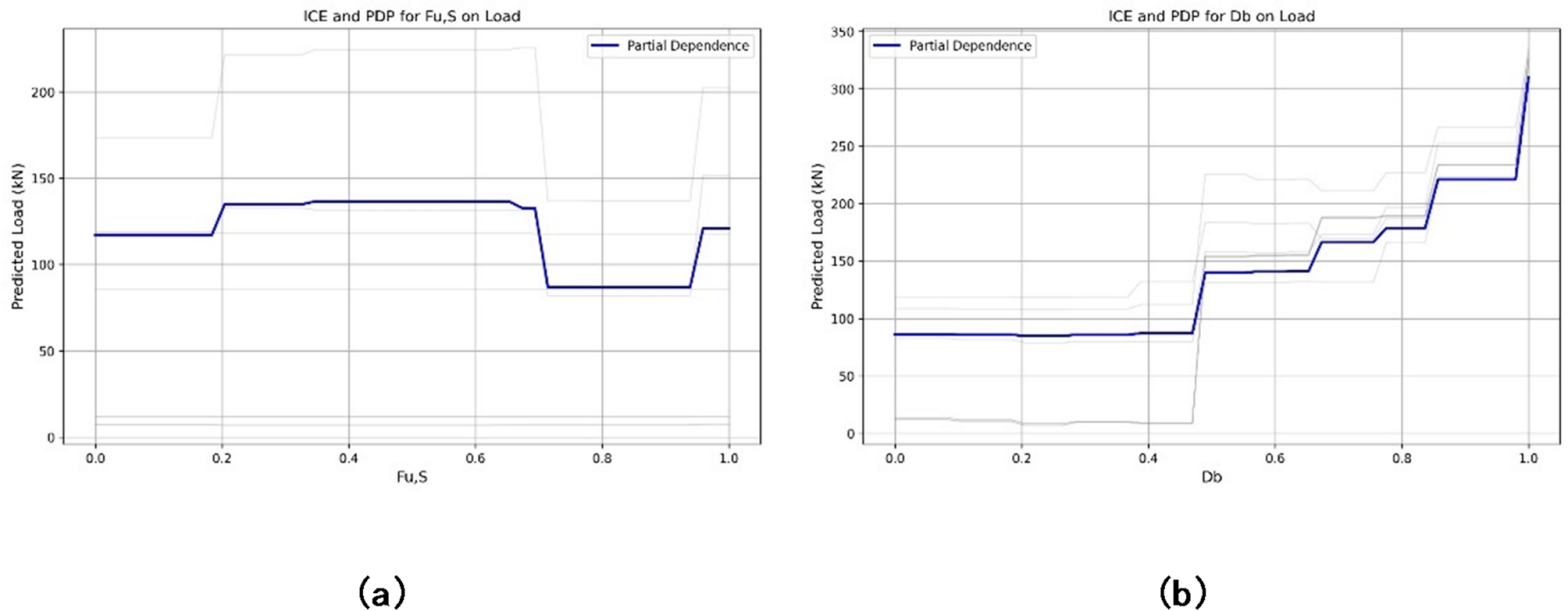
The partial dependance plots for: (**a**) steel ultimate strength, F_u, S_​ (**b**) bolt diameter, D_b_ on load.

Figure 11 shows the partial dependence plots (PDPs) for D_b_ and F_u_S_ on load provide critical insights into their non-linear relationships with the predicted outcome. The PDP for D_b_ shows a relatively stable predicted load (around 100 kN) up to 0.6, followed by a sharp increase to approximately 350 kN as D_b_ approaches 1.0, suggesting a threshold effect where higher D_b_ values significantly boost load predictions. In contrast, the PDP for F_u_S_ exhibits a step-wise pattern, with load remaining constant at around 100 kN until 0.2, then increasing to 150 kN between 0.2 and 0.6, and further rising to 200 kN beyond 0.6, indicating discrete shifts in influence. These patterns highlight D_b_ and F_u_S_ as pivotal features, with their impact varying across ranges, enhancing model interpretability.

## Scope of the developed ML models

A varied dataset reflecting realistic structural configurations and material parameters was gathered in order to guarantee the accuracy and generalizability of machine learning (ML) models for forecasting the final load capacity of demountable shear connectors. The chosen characteristics cover a broad spectrum of mechanical, geometric, and interfacial circumstances that are frequently present in composite systems made of steel and concrete. These input variables, which capture the intricate relationships between steel, concrete, grout, and connector components, were selected based on engineering judgment and experimental significance. The dataset facilitates accurate prediction across a range of loading scenarios and robust model training by encompassing the whole spectrum of practical values as the following:

- Compressive strength of reinforced concrete: 23.06 MPa ≤ *F*_*cu, C*_ ≤ 65 MPa.
- Compressive strength of grout: 0 MPa ≤ *F*_*cu, G*_ ≤ 95.1 MPa.
- Yield strength of steel: 235 MPa ≤ *F*_*y, S​*_ ≤ 375 MPa.
- Ultimate strength of steel: 350 MPa ≤ *F*_*u, S*_​ ≤ 541 MPa.
- Bolt diameter: 6 mm ≤ *D*_*b*_ ≤ 27 mm.
- Number of bolts: 4 ≤ *N* ≤ 8.
- Yield strength of bolts: 248.81 MPa ≤ *F*_*y, b*_ ≤ 1115 MPa.
- Ultimate strength of bolts: 385.8 MPa ≤ *F*_*u, b*_ ≤ 1319 MPa.
- Bolt pretension force: 0 kN ≤ *T*_*b*_​ ≤ 155 kN.
- Friction coefficients for all interfaces (e.g., beam–concrete, bolt–grout, nut–beam): 0 ≤ *f* ≤ 0.7.

## Interactive software development for shear capacity prediction

**Fig. 12:**
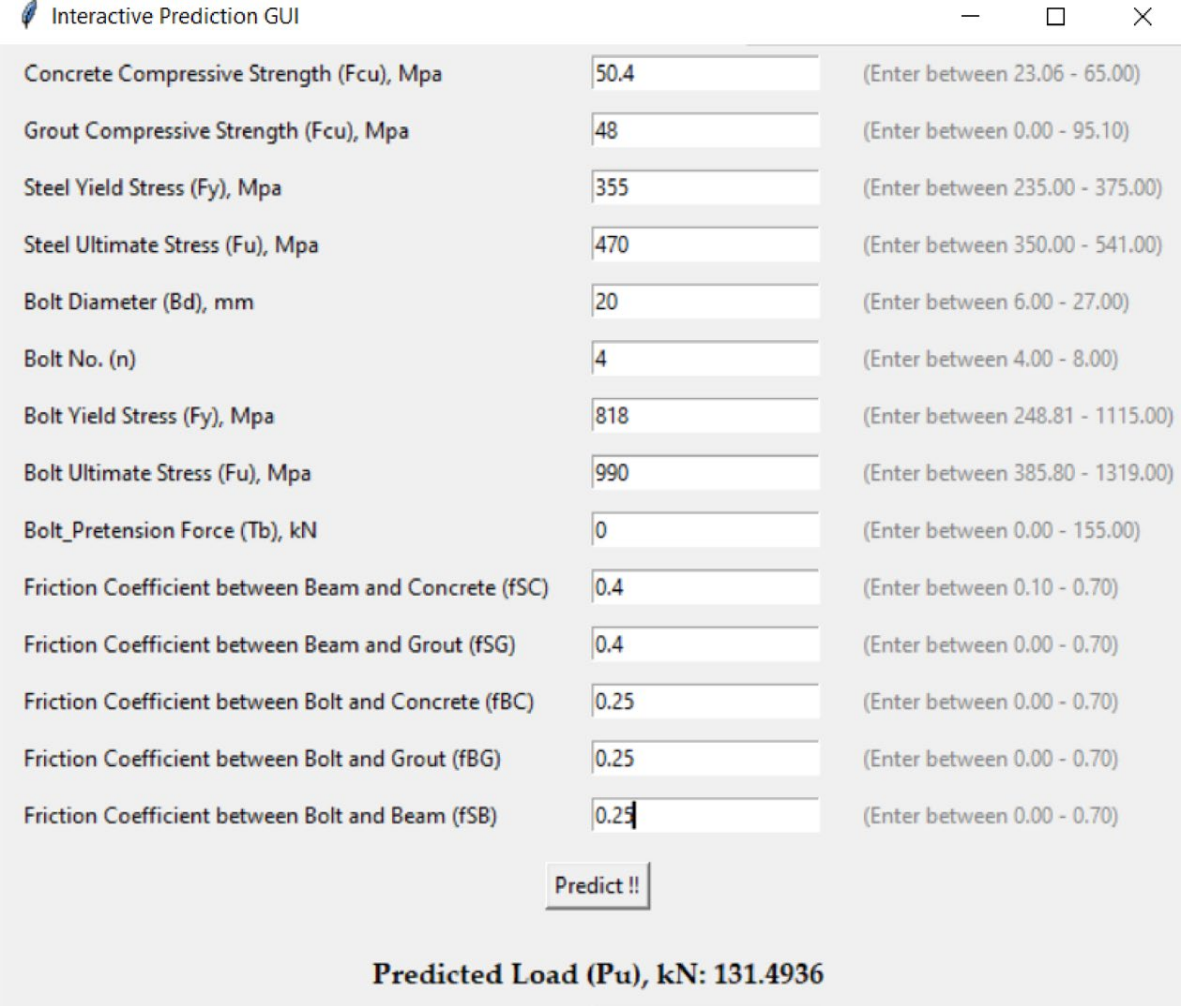
Interface of developed interactive software.

To translate the outcomes of this research into a practical tool for engineers, an interactive software application was developed to predict the ultimate shear capacity of demountable shear connectors. The program was written in Python^45^built around the two top-performing machine learning models XGBoost and Random Forest which demonstrated high predictive accuracy, with *R²* values of 0.9477 and 0.9255, respectively. As shown in Fig. 12, the software features an intuitive and user-friendly interface that allows users to input key design variables, such as bolt diameter, concrete and grout strength, bolt material properties, and interface friction coefficients. Once the data is entered, the software instantly calculates the predicted shear capacity and displays the results along with a visual explanation of feature contributions using SHAP values. This functionality not only supports accurate estimation but also helps users understand the impact of each input on the final outcome. Designed for accessibility and efficiency, the tool bridges the gap between complex machine learning techniques and practical engineering application, enabling faster, smarter, and more transparent decision-making in the design of demountable shear stud connectors.

## Conclusion

This study highlights the potential of machine learning (ML) techniques in accurately predicting the ultimate load capacity of demountable shear connectors in steel–concrete composite structures. By training and evaluating eight supervised regression models on a diverse dataset of 239 experimentally derived samples, the research provided meaningful insights into both model performance and the role of key design variables in structural behavior.

- Among all models, XGBoost and Random Forest demonstrated the highest predictive capabilities, achieving *R²* values of 0.9477 and 0.9255, respectively. These ensemble learning methods consistently outperformed traditional linear models such as Linear Regression, Ridge, and Lasso, as well as Support Vector Regression (SVR), which showed the weakest performance across all evaluation metrics. The superior accuracy of ensemble models is largely due to their ability to model complex, nonlinear relationships within the dataset.
- To enhance model interpretability, SHAP analysis was employed to evaluate the contribution of individual features. The results identified bolt diameter, steel ultimate strength, and bolt-to-concrete friction as the most influential parameters affecting load prediction. Conversely, variables such as grout strength, bolt pretension, and certain friction coefficients involving beam and grout showed minimal impact, suggesting these may be simplified or deemphasized in future design-focused models.
- The findings were further supported by feature importance heatmaps and true vs. predicted scatter plots, which visually reinforced the reliability of the top-performing models.
- Despite these promising outcomes, some challenges remain. The dataset, although extensive, may still lack representation of extreme or failure-specific behaviors under diverse environmental or loading conditions. Additionally, multicollinearity among certain input features especially friction parameters can affect model generalizability.
- Future research should aim to enrich the dataset through additional experiments and simulations, incorporate dynamic or cyclic loading conditions, environmental degradation, or time dependent behaviour and explore hybrid models that combine physics-based and data-driven approaches. In addition, we could add some parameters as slip modulus, ductility index, or failure mode classifications.

## Supplementary Information

Below is the link to the electronic supplementary material.

Supplementary Material 1

## Data Availability

All data generated or analyzed during this study are included in this published article as supplementary information files. In addition, any additional required data is available by the corresponding author.
